# Persistent Methicillin-Resistant Staphylococcus Aureus Bacteremia Secondary to Infected Seroma: A Rare Case Report

**DOI:** 10.7759/cureus.22390

**Published:** 2022-02-19

**Authors:** Ruhma Ali, Aditya Patel, Ahmed Abbas, Muhammad Hussain, Jihad Slim, Jack Boghossian

**Affiliations:** 1 Internal Medicine, Saint Michael's Medical Center, Newark, USA; 2 Infectious Diseases, Saint Michael's Medical Center, Newark, USA

**Keywords:** septic emboli, source control, methicillin resistant staphylococcus aureus, bacteremia, seroma

## Abstract

A seroma is defined as a serous fluid collection that develops as a response to injury and surgeries, particularly mastectomy and reconstructive and abdominal surgeries. The majority of the seromas are self-limiting and arise in the acute postoperative period; however, diagnosis of seroma several years after surgery has also been reported in the literature. Persistent bacteremia with infected seroma as a source is a rare entity. We present the first case to be reported of persistent bacteremia secondary to infected seroma with septic emboli to lungs and prostate without any evidence of endocarditis on multiple echocardiograms. This case highlights the importance of meticulous physical examination and source control in the management of bacteremia.

## Introduction

A seroma is a serous fluid collection that builds up in a surgical dead space following skin flap formation [1]. The fluid is composed of inflammatory products produced by the injured cells during surgery and the plasma from the damaged lymphatic channels [2]. Seromas are particularly common after mastectomy and abdominal and reconstructive surgeries [3]. The dead space leads to the prevention of tissue adhesion by the accumulation of lymphatic, exudative, and inflammatory fluids [4]. Several factors have been implicated in the formation of seroma, including age, comorbid conditions, and the extent of lymph node clearance. There are various options to treat the condition, including percutaneous aspiration, external compression, sclerotherapy, and additional surgical intervention [5]. Seroma can become infected by the seeding of bacteria during percutaneous drainage. However, to the best of our knowledge, the presence of persistent bacteremia due to an infected seroma has not been reported in the literature so far. We report a case of persistent methicillin-resistant *Staphylococcus aureus* (MRSA) bacteremia with septic emboli to the lungs and prostate secondary to infected neck seroma in a patient with poorly controlled diabetes.

## Case presentation

A 59-year-old male with a past medical history of diabetes mellitus, peripheral neuropathy, and hypertension presented to the emergency department with complaints of generalized fatigue, malaise, and fever for the past one week. He also complained of dribbling of urine for the past few days. He denied dysuria, suprapubic pain, or perineal pain. He reported sexual encounters with two partners in the last six months without the use of a condom but denied any complaint of penile discharge, intravenous drug abuse (IVDU), any history of sexually transmitted diseases, or indwelling prosthesis. He was up to date with his vaccinations including the coronavirus disease 2019 (COVID-19) vaccine. He denied chills, chest pain, shortness of breath, palpitations, nausea, vomiting, diarrhea, and abdominal pain or weight loss. A review of the system was negative except for the complaints mentioned above.

The patient had a history of abscess in the nape of the neck three months earlier, which had been debrided at another hospital and treated with antibiotics. He did not remember the name and duration of the antibiotic regimen. His diabetes and hypertension were poorly controlled and he admitted to being non-compliant with medications.

On admission, the patient was febrile with a maximum temperature of 100.7 °F, mildly tachypneic with a respiratory rate of 25 breaths/minute; he had a pulse rate of 81 beats/minute, blood pressure of 125/70 mmHg, and he was saturating at 97% on a 3-L nasal cannula and 94% on room air (RA). His BMI was 32.3 kg/m^2^. On physical examination, he was awake, alert, and oriented. Localized, nonerythematous, non-tender, and non-fluctuant swelling was noticed on the nape of the neck. The lungs were clear to auscultation bilaterally. No wheezing, rhonchi, or rales were heard. Cardiac examination was normal without any murmurs, rubs, or gallops. The abdomen was soft and non-distended with normal bowel sounds. No petechiae and rashes were noted on the skin and fingernails. The remainder of the physical examination was within normal limits. Initial laboratory examination was significant for leukocytosis with an elevated absolute neutrophil count. His C-reactive protein, procalcitonin, and lactic acid levels were elevated. The antigen and polymerase chain reaction (PCR) for severe acute respiratory syndrome coronavirus 2 (SARS-CoV-2) were negative. D-dimer was significantly elevated, and he had high anion gap metabolic acidosis with hyperglycemia. The lab findings are presented in Table 1.

**Table 1:** Laboratory parameters

Laboratory parameters	Values	Reference range
Sodium	123	136–145 mmol/L
Potassium	4.5	3.5–5.3 mmol/L
Chloride	85	98–110 mmol/L
Blood urea nitrogen (BUN)	23	6–24 mg/dL
Creatinine	1.4	0.6–1.2 mg/dL
Corrected anion gap	19.3	5–15 mmol/L
Glucose	434	70–140 mg/dL
D-dimer	2,731	0–500 ng/mL
White blood cells (WBC)	19	4.4–11 x10^3^/uL
Absolute neutrophil count (ANC)	18.1	1.7–7 x10^3^/uL
Hemoglobin	14.4	13.5–17.5 g/dL
Platelets	404	150–450 x10^3^/uL
Blood pH	7.45	7.31–7.45
C-reactive protein (CRP)	25.9	0–0.8 mg/dL
Procalcitonin	8.91	0–0.5 ng/mL
Lactic acid	4.6	0–2 mmol/L
Prostate-specific antigen	6.5	0–4 ng/ml
Hemoglobin A1c	12.7	4–5.6%

**Figure 1:**
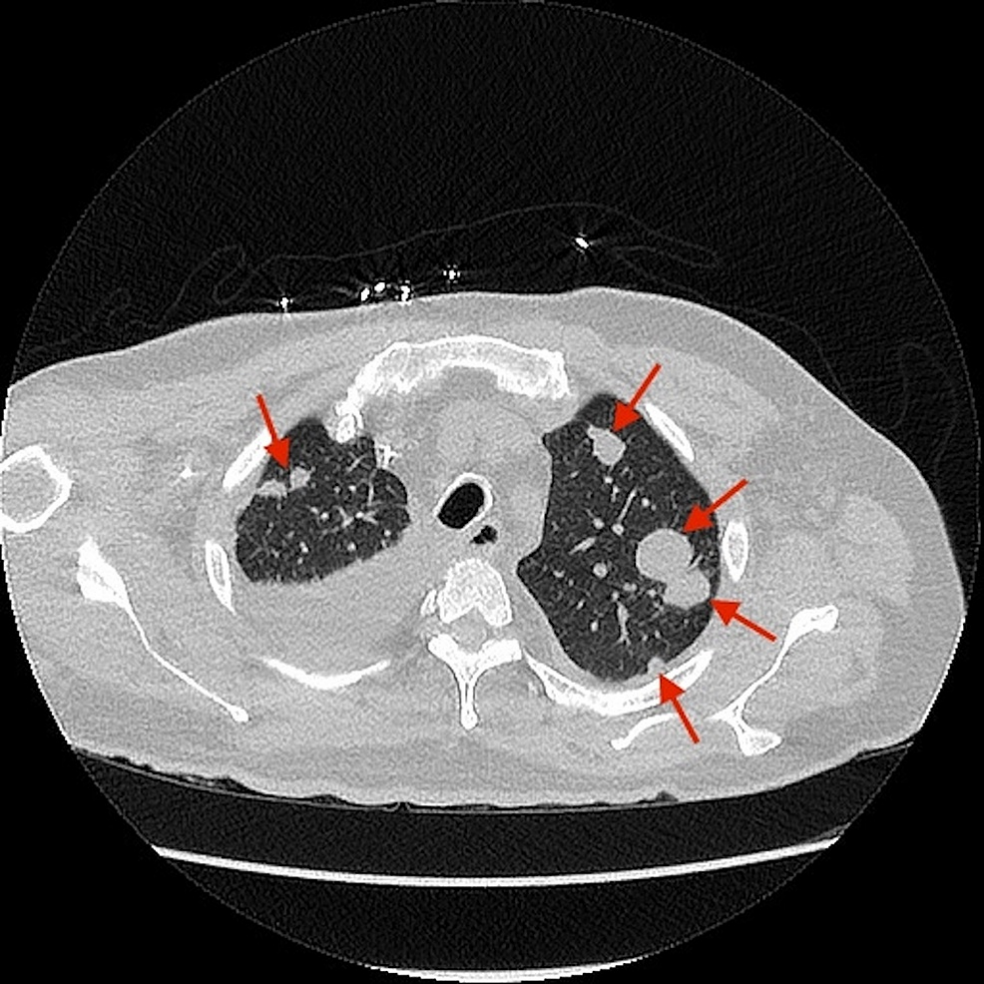
CT scan of the chest with contrast showing multifocal infiltrates throughout the lungs (arrows) CT: computed tomography

**Figure 2:**
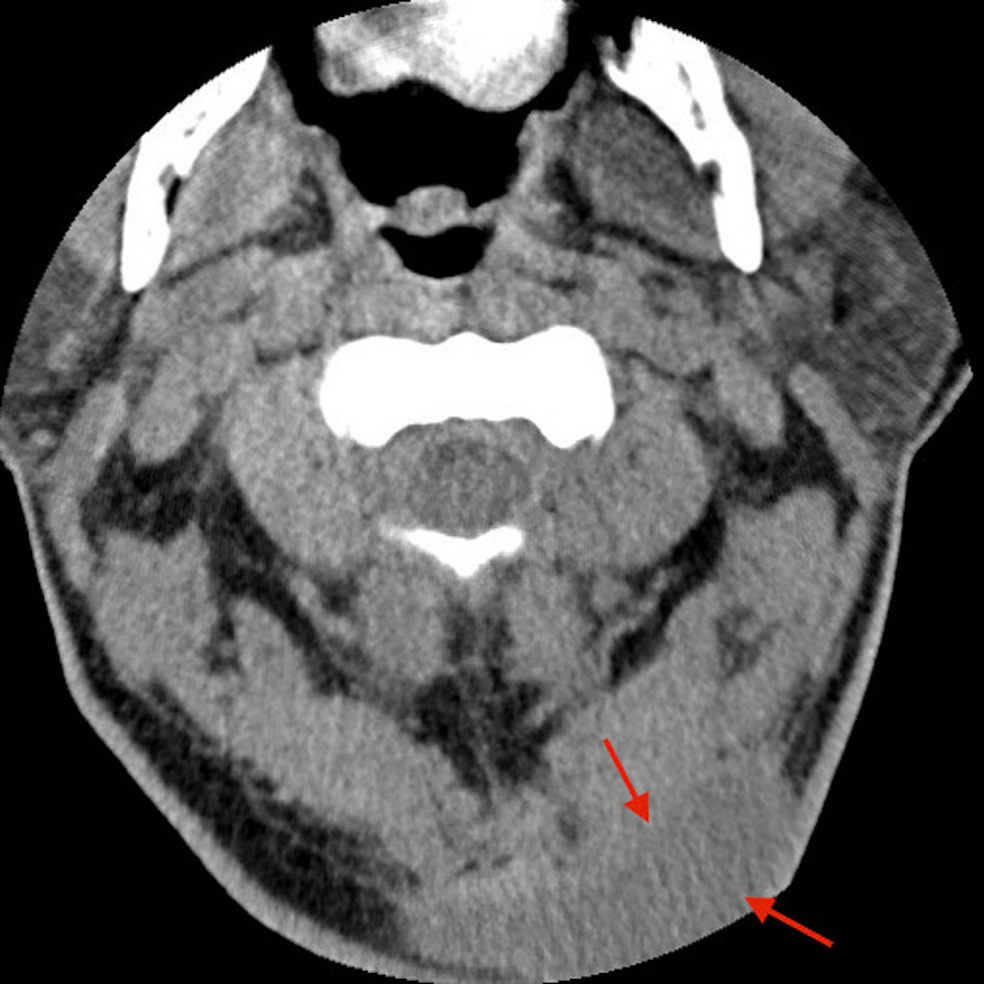
CT cervical spine without contrast showing large abnormality in the posterior subcutaneous region of the upper cervical spine at the midline and towards the left (arrows) CT: computed tomography

The patient was admitted to the ICU for diabetic ketoacidosis (DKA) and severe sepsis. The rapid plasma reagent was non-reactive. The nucleic acid amplification tests for *Chlamydia trachomatis* and *Neisseria gonorrhoeae* were negative. The HIV antibody test was non-reactive, and EKG showed normal sinus rhythm. The chest X-ray showed multifocal infiltrates. CT scan of the abdomen and pelvis showed enlarged prostate glands with multiple regions of hypoattenuation. CT scan of the chest showed multiple nodular opacities throughout the lungs with dense basilar consolidation bilaterally as shown in Figure 1. The CT cervical spine without contrast showed a large abnormality in the posterior subcutaneous region of the upper cervical spine at the midline and towards the left as shown in Figure 2. CT lumbar and thoracic spine were unremarkable. MRI of the cervical spine without contrast showed a fluid collection in the suboccipital region on the left consistent with an abscess.

The patient was started on DKA protocol; empiric vancomycin and cefepime were given for the differential diagnosis of infective endocarditis. The blood and urine cultures were positive for MRSA, and the antibiotics were deescalated to vancomycin and cefazolin. Incision and drainage (I&D) of the fluid collection in the suboccipital region of the neck was done. The fluid culture from the collection was also positive for MRSA. Four blood cultures done over the next seven days were persistently positive until infected seroma was drained. Transthoracic echocardiography (TTE) was done to rule out endocarditis, which showed no vegetations. The patient continued to have persistent MRSA bacteremia for a week with negative TTE and transesophageal echocardiogram (TEE). A diagnosis of persistent MRSA bacteremia secondary to infected seroma was made; however, the presence of multiple nodules in the lungs and hypoattenuation in the prostate was unusual without any evidence of vegetation on the echocardiogram. The patient was switched to linezolid after 17 days and TTE was repeated on day 14 with no evidence of vegetation, and a repeat CT chest showed improvement in pulmonary nodules. The patient was discharged on linezolid with outpatient follow-up with the infectious disease service. He completed six weeks of antibiotics therapy with clinical improvement and normalization of inflammatory markers and prostate-specific antigen (PSA).

## Discussion

A seroma is a pocket of clear fluid that develops after surgery, particularly mastectomy and axillary surgery, or injury [6]. The reported incidence of seroma formation after mastectomy ranges between 15-81% [7]. The incidence of seroma after ventral hernia repair is approximately 30% [8]. Seroma associated with the thyroid and parathyroid surgeries has an incidence of 0-6% and depends on the extent of the surgeries [9]. There is no consensus on the definition of seroma in the published literature; some report a seroma when a patient is symptomatic while other physicians define seroma as a fluid collection of 15-20 ml [10]. The wide range in the rate of incidence might be attributed to the varying methods of defining seroma in the literature. The majority of the seromas are self-limiting and resolve spontaneously [11]. Many theories have been proposed regarding the pathophysiology of seroma formation. Seroma has an acute inflammatory component in response to surgical trauma due to the activities of cytokines, growth factors, proteinases, and proteinase inhibitors [12]. Another hypothesis puts forward extensive tissue dissection with the formation of a dead space for the granulation tissue and fluid to accumulate. Seroma fluid is exudative with similar characteristics as those of lymph in the early postoperative period [13]. Oertli et al. suggested that fibrinolytic activity was involved in seroma formation [14].

A prospective randomized trial by Petrek et al. showed that the number and extent of axillary lymph node involvement was the most significant influencing factor in the occurrence of seroma [15], whereas Hashemi et al. reported that the type of surgery was the only significant factor in the formation of seroma [16]. In our case, the seroma developed after I&D of a neck abscess, which was later on infected and complicated by MRSA bacteremia with septic emboli without any evidence of vegetation on multiple echocardiograms. This case is unique because seroma has not been previously implicated as the source of persistent bacteremia in the literature. Imaging techniques are the modality of choice for the diagnosis of seroma. Seroma may cause pain, cosmetic disfigurement, and functional limitation depending on the area of the body involved. Ultrasound and MRI can be employed for a precise assessment. Several techniques have been described for the prevention and reduction of seroma, including compression dressing, use of drains, and sclerotherapy; however, no single method has been shown to be consistently effective [10].

The mainstay of treatment of infected seroma includes systemic antibiotics and drainage, whereas further surgical intervention is required if conservative management is unsuccessful or in cases of recurrent seromas. The use of sclerotherapy for the management of seroma has also been reported with minimal complications [2]. Some studies have also reported the successful use of topical tetracycline in the solution form for the management of seroma due to its sclerosing effect [17]. In addition, the use of doxycycline provides local delivery of an antibiotic commonly used for skin and soft tissue infections due to its efficacy against *Staphylococcus aureus*, particularly MRSA. Definitive treatment will vary according to the patient characteristics. In our case, the patient’s symptoms improved with systemic antibiotics and no recurrence of the seroma was noted. This case also highlights the importance of meticulous physical examination to identify the source of septicemia in order to adequately treat the infection and prevent future recurrence. It also emphasizes the need for source control when encountering persistent bacteremia, since our patient's bacteremia resolved after I&D.

## Conclusions

This case suggests that a high index of suspicion for seroma being the source of bacteremia should be maintained for patients with persistent symptoms. To the best of our knowledge, this is the first case in the literature about bacteremia with septic emboli secondary to infected seroma in a diabetic patient without any evidence of vegetations on multiple echocardiograms. Our patient's condition improved with antibiotics and source control (I&D).
